# Impacts of 25 years of groundwater extraction on subsidence in the Mekong delta, Vietnam

**DOI:** 10.1088/1748-9326/aa7146

**Published:** 2017

**Authors:** P S J Minderhoud, G Erkens, V H Pham, V T Bui, L Erban, H Kooi, E Stouthamer

**Affiliations:** 1 Department of Physical Geography, Utrecht University, P.O. Box 80115, 3508 TC Utrecht, The Netherlands; 2 Unit of Soil and Groundwater Systems, Deltares, Delta Research Institute, P.O. Box 85467, 3508 AL, Utrecht, The Netherlands; 3 Division of Water Resources Planning and Investigation for the South of Vietnam (DWRPIS), Ho Chi Minh City, Vietnam; 4 US EPA Office of Research and Development, National Health and Environmental Effects Research Laboratory, Atlantic Ecology Division, Narragansett, RI, United States of America; 5 Author to whom any correspondence should be addressed.

**Keywords:** Delta subsidence, sea-level rise, groundwater exploitation, modelling, Imod

## Abstract

Many major river deltas in the world are subsiding and consequently become increasingly vulnerable to flooding and storm surges, salinization and permanent inundation. For the Mekong Delta, annual subsidence rates up to several centimetres have been reported. Excessive groundwater extraction is suggested as the main driver. As groundwater levels drop, subsidence is induced through aquifer compaction. Over the past 25 years, groundwater exploitation has increased dramatically, transforming the delta from an almost undisturbed hydrogeological state to a situation with increasing aquifer depletion. Yet the exact contribution of groundwater exploitation to subsidence in the Mekong delta has remained unknown. In this study we deployed a delta-wide modelling approach, comprising a 3D hydrogeological model with an integrated subsidence module. This provides a quantitative spatially-explicit assessment of groundwater extraction-induced subsidence for the entire Mekong delta since the start of widespread overexploitation of the groundwater reserves. We find that subsidence related to groundwater extraction has gradually increased in the past decades with highest sinking rates at present. During the past 25 years, the delta sank on average ∼18 cm as a consequence of groundwater withdrawal. Current average subsidence rates due to groundwater extraction in our best estimate model amount to 1.1 cm yr^−1^, with areas subsiding over 2.5 cm yr^−1^, outpacing global sea level rise almost by an order of magnitude. Given the increasing trends in groundwater demand in the delta, the current rates are likely to increase in the near future.

## 1. Introduction

The low-lying and densely populated Mekong delta (MKD), largely located in Vietnam, has the third largest delta plain in the world (Coleman and Roberts 1989). The delta is fertile, intensively cultivated, and responsible for 50% of Vietnam’s total food production. As Vietnam is the world’s second largest rice exporter, and 90% of all rice is produced in the MKD, over 200 million people rely on the delta for food (GSO 2016).

By nature, low-lying delta systems are sensitive to environmental change. The MKD is threatened by global sea-level rise (Wassmann et al 2004), natural decrease in fluvial sediment supply (Darby et al 2016), enhanced by upstream sediment trapping behind dams (Kondolf et al 2014, Minh et al 2015), salinization (Renaud et al 2015) and coastal erosion (Anthony et al 2015). On top of that, highly compressible soils make deltas worldwide vulnerable to subsidence (Syvitski et al 2009), increasing relative sea-level rise (RSLR). In the MKD subsidence rates seem to exceed global eustatic SLR by an order of magnitude (Erban et al 2014). As a result, subsidence acts as a catalyst, increasing vulnerability to flooding and storm surges, saltwater intrusion in the channels and risk of permanent inundation of the delta. Groundwater overexploitation has been proposed to be the main driver of subsidence in the MKD (Erban et al 2013, 2014), corresponding to observations in other subsiding deltas and coastal areas around the world (Phien-wej et al 2006, Teatini et al 2006, Saito et al 2007, Abidin et al 2011, Higgins et al 2013). In the MKD, the demand for fresh water has steadily risen following the ongoing economic growth after Vietnam’s transition to a market economy in 1986. This transition stimulated massive cultivation, urbanisation and industrialisation in the MKD (Seto 2011). With surface water often being polluted or saline, groundwater is the main source to meet this increasing freshwater demand (Wagner et al 2012). In 1991, when groundwater monitoring in the MKD commenced, piezometric levels in the aquifer system were at more or less natural levels (i.e. at or above delta surface elevation) in most parts of the delta. Over the past 25 years, groundwater exploitation strongly increased, resulting in a persistent drawdown of hydraulic heads (i.e. water pressure) throughout the entire delta subsurface. This process is known to trigger fine-grained sediment consolidation in the subsurface, causing aquifer-system compaction (e.g. Galloway and Burbey 2011, Gambolati and Teatini 2015), expressed as land subsidence of the delta surface.

Average subsidence rates for the Mekong delta were determined at 6 mm yr^−1^ from 1987–2006 by surface water level trend analysis (Fujihara et al 2015), 17.1 mm yr^−1^ from 1993–2013 for Can Tho city (Takagi et al 2016) and 16 mm yr^−1^ from 2006–2010 at 15 monitoring stations by InSAR (Interferometric Synthetic Aperture Radar) (Erban et al 2014). For Ho Chi Minh City (HCMC) local extreme rates of 46 mm yr^−1^ (Erban et al 2014), and 70 mm yr^−1^ (Minh et al 2015) are reported. As InSAR is unable to measure areas lacking clear reflectors, such as rural areas that constitute most of the MKD, the analyses only cover part of the delta. Local 1D subsidence calculations as a function of measured groundwater drawdown have been performed for point locations in the MKD (Erban et al 2014), Ca Mau city (Karlsrud and Vangelsten 2017) and HCMC (Thoang and Giao 2015). Interpolation maps based on such subsidence calculations from sparse groundwater monitoring well locations fail to reproduce the actual situation, since factors that influence subsidence locally, such as spatial heterogeneity of the delta subsurface and variability in the hydrogeological situation, are unaccounted for. Consequently, a delta-wide cumulative subsidence map of the MKD still does not exist. Furthermore, the relative contribution of 25 years of groundwater extraction to the total observed subsidence rates is unknown, as well as the current rates of groundwater extraction-induced subsidence.

In this paper, we present the first delta-wide quantification of groundwater extraction-induced subsidence over the last 25 years in the MKD. Using a newly developed 3D numerical groundwater flow model of the delta subsurface, we simulated ground-water drawdowns based on measured time series of hydraulic heads and an estimate of the extraction history. Subsequently, we calculated the corresponding aquifer system consolidation using a one-way coupled subsidence module. Our approach enables the evaluation of groundwater extraction-induced subsidence at delta scale. The modelling period captures the onset of widespread groundwater drawdown, allowing us to quantify the evolution of subsidence rates and total cumulative subsidence due to groundwater extraction over the past decades, pre-dating measurements, until present. Our process-based modelling approach provides an important step towards disentangling the measured total subsidence signal into the relative contribution of different natural and human-induced drivers to total subsidence for deltaic areas like the MKD. This will greatly benefit thorough and knowledge-based predictions of delta-wide subsidence for the coming decades, supporting urgently needed decision-making in subsiding deltas (Galloway et al 2016).

## 2. Model setup and calibration

We created a delta-wide, 3D hydrogeological ground-water model for a physical, process-based interpolation of measured drawdown rates in time and space driven by groundwater extraction. Groundwater flow was modelled using the MODFLOW-based environment iMOD (Vermeulen 2006, Minnema et al 2013). The lowering of water pressure in the subsurface was subsequently used to calculate subsidence in the entire aquifer system using a one-way coupled geotechnical subsidence module called SUB-CR (Kooi et al submitted). The main focus of the hydrogeological model was to simulate the evolution of relative hydraulic head (i.e. water pressure) changes, driving subsidence. Absolute head levels are therefore less relevant for this study. Vermeulen et al (2013) built a first quasi-3D, steady-state groundwater model of the MKD in iMOD. To be able to simulate subsidence and meet our model requirements, we built a new transient model in which confining layers are explicitly modelled. The model was based on available geological, hydrological and geotechnical data, supplied by the Division of Water Resources Planning and Investigation for the South of Vietnam (DWRPIS).

### 2.1. Hydrogeological model

**Figure 1.**
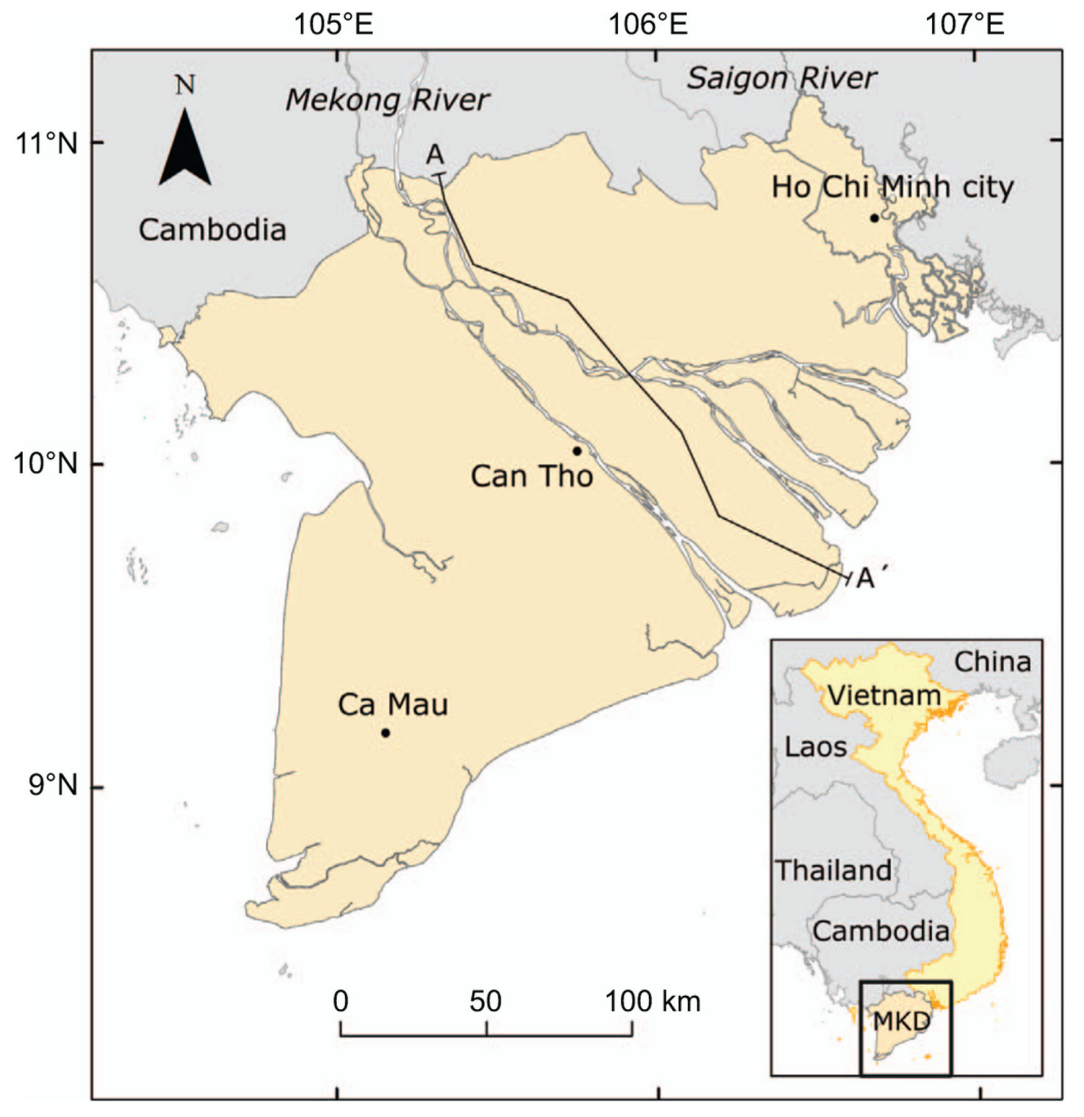
Location of the Mekong Delta and part of the interconnected Saigon River to the northeast, encompassing the province of Ho Chi Minh City, in the south of Vietnam. The map extent corresponds with the model extent. Cross-section line A-A’ shows the location of the hydrogeological cross-section in figure 2.

A numerical hydrogeological model of the entire MKD and part of the inter-connected Saigon river delta, hosting HCMC, was constructed (figure 1). A 3D model of the aquifer-aquitard subsurface was built using the iMOD SolidTool (Vermeulen et al 2016) by interpolating 95 borehole logs in ten hydrogeological cross-sections, dividing the delta subsurface into seven main hydrogeological units distinguished by the Division for Geological Mapping for the South of Vietnam (DGMS 2004, figure 2). This resulted in a 15 layer, subsurface model, representing seven aquifers, seven aquitards and a phreatic top layer. See the supplementary information (S1) available at stacks.iop.org/ERL/12/064006/mmedia for a more in-depth description of the subsurface model.

The 3D schematization of the MKD subsurface was used to build a transient hydrogeological model at 1 × 1 km^2^ horizontal resolution to simulate groundwater flow and fluctuations in hydraulic head over the past 25 years (1991–2015) with monthly time steps. The boundary of the active model area was positioned 20 km outside the national border and 50 km off-shore to account for lateral groundwater flow in the modelled delta, i.e. trans-boundary flow from Cambodia (Erban and Gorelick 2016) and off-shore groundwater flow (e.g. Post et al 2013). Recharge is modelled based on annual amounts of precipitation and evaporation measured from 1999–2010. The amounts were spatially modelled using distribution maps (Luong 2008), and for each month the average, multi-year, monthly percentage was taken. The average measured values of precipitation and evaporation were assigned for the modelling period beyond the measurement records. The surface water network in the MKD was not explicitly modelled. Recharge from the river system to the aquifers in the down stream part of the MKD is expected to be limited due to the presence of the thick, largely impermeable, Holocene aquitard near the surface effectively sealing off the aquifers below, and therefore was not considered in the model. A model run using a simplified river system of the Mekong river from Vermeulenetal(2013)confirmedthisassumptionasthe inclusion of the river system only affected calculated subsidence significantly for some model cells located along the river system. Drainage was modelled by a constant drain level 0.5 m below surface level, simulating the draining effect of the dense network of paddies, surface channels and canals cross-cutting the delta. Initial hydraulic heads followed from a steady-state simulation without groundwater extraction and average recharge values (see online supplementary S3 for a summary of the model setup).

**Figure 3.**
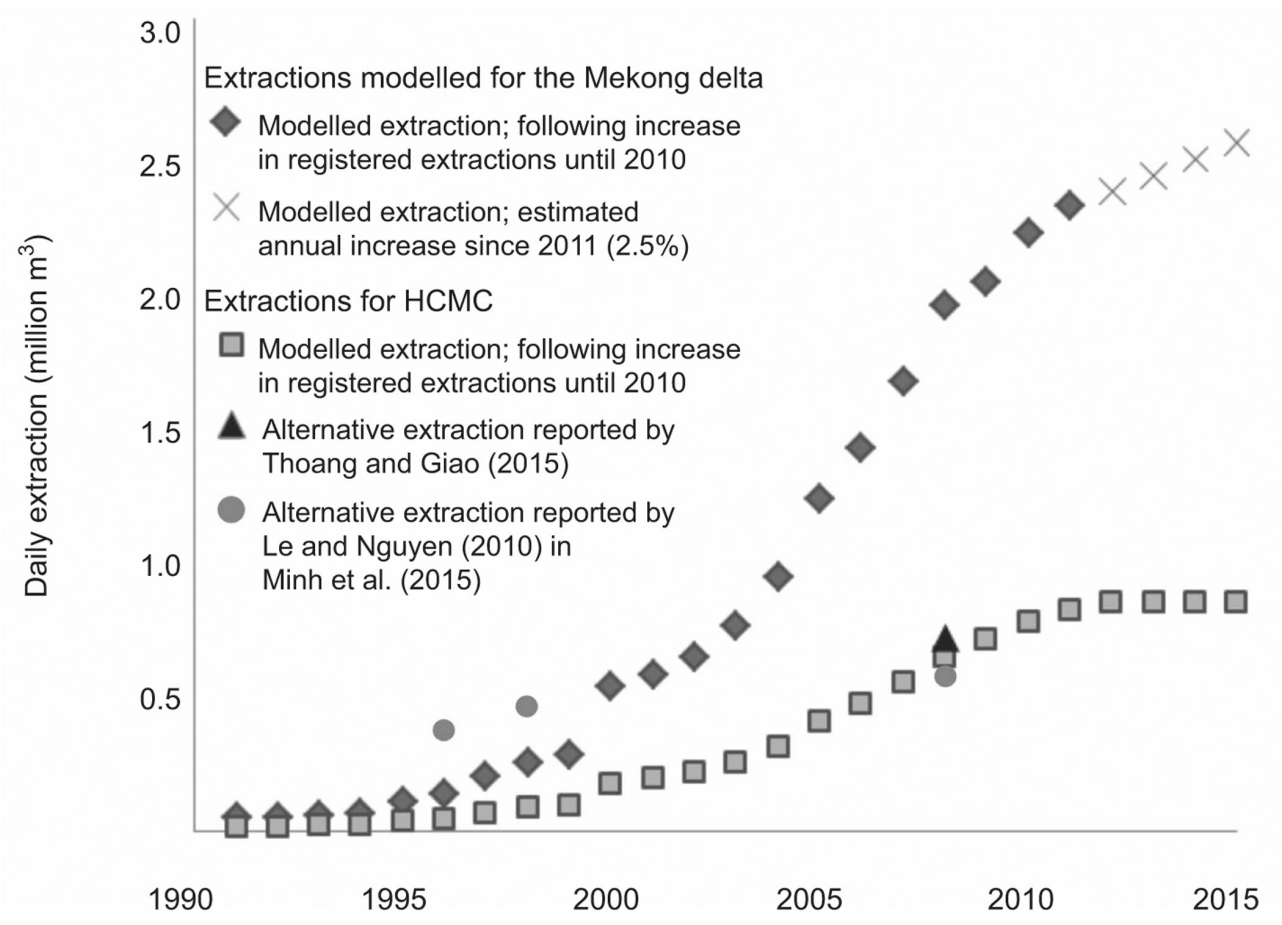
Annual modelled groundwater extraction in the MKD and HCMC province. An annual increase of 2.5% is assumed for extraction in the MKD after 2011. The dots (Le and Nguyen 2010 in Minh et al 2015) and triangle (Thoang and Giao 2015) show alternative values reported for HCMC province.

Groundwater extraction during the transient modelling period of the past 25 years was modelled based on an integration of several datasets reporting extracted volumes for the MKD and the HCMC province (DWRPIS 2010)(S3). In total extraction in the MKD was modelled at over 15 000 locations, including large wells and clusters of small household wells, accounting for a daily abstracted volume of over 2.5 million m^3^ (figure 3). For HCMC province more than 1300 well nests are responsible for extracting over 800 000 m^3^ on daily basis. As a result of a water act in HCMC to restrict groundwater exploitation (HCMC 2007), the extracted volume in HCMC has gradually stabilised in recent years.

Initial hydrogeological model parameterization for the MKD was based on available data of the DWRPIS (SI). The hydraulic conductivity (K_h_) and storage coefficient (SS_c_) of each model layer were calibrated through an automated parameter estimation (PEST) protocol (following the approach of Olsthoorn (1995), described in Vermeulen et al 2016) using piezometric head measurements from 91 monitoring wells located throughout the delta and HCMC (see online supplementary S4 for description and calibrated parameter values).

### 2.2. Subsidence calculation and parameterization

Subsidence due to aquifer-system compaction following the hydraulic head decline (i.e. decreasing pressure) was calculated using the abc model (Den Haan 1994). This model determines natural strain (i.e. degree of compression) based on the isotach concept first proposed by Šuklje (1957) and extended by Bjerrum (1967). In this model natural strain (ε^H^) is described as follows:

(1) $$\varepsilon^{H}=\int_{m_{0}}^{m_{\text{final}}}\frac{1}{m}dm$$

where *m* is momentary layer thickness. The model decomposes total strain (*ε*^*H*^) into two components, a direct elastic contribution $\left(\varepsilon_{d}^{H}\right)$ and a time-dependent (secular) creep contribution $\left(\varepsilon_{s}^{H}\right)$; *$\varepsilon^{H}=\varepsilon_{d}^{H}+\varepsilon_{s}^{H}$* The first component accounts for the elastic (i.e. reversible) response to changes in effective stress, and the latter component for the permanent strain that develops by creep (viscous deformation). Creep, which is widely used in geotechnical models of land surface settlement induced by surface loads, is considered more appropriate to model the deformation of clay and peat than plastic deformation that is more commonly employed in land subsidence models (e.g. SUB-WT; Leake and Galloway 2007) for secondary consolidation is ignored in these models. Three compression parameters define the system of the *abc* method: *a* (recompression or swelling constant) accounts for the elastic compression, *b* (compression constant) and *c* (secondary compression constant) for the visco-plastic compression. Using these constants, total natural strain is calculated as a function of effective stress and intrinsic time (τ) by the following expression (Den Haan 1994):

(2) $$\varepsilon^{H}=a\;\ln\left(\frac{{\sigma^{\prime }}_{\text{p}}}{{\sigma^{\prime }}_{o}}\right)+b\;\ln\left(\frac{\sigma^{\prime }}{{\sigma^{\prime }}_{\text{p}}}\right)+c\;\ln\left(\frac{\tau }{\tau_{\text{ref}}}\right)$$

where σ′_p_ is the initial pre-consolidation stress,σ′_o_ the initial effective stress, σ^′^ the momentary effective stress and τ_ref_ the reference time (=1 day). Intrinsic time (τ)is calculated as follows:

(3) $$\tau =\tau_{\text{ref}}{\text{OCR}}^{\frac{b-a}{c}}$$

where *a*, *b* and *c* are the compression parameters and OCR is overconsolidation ratio which is described by this general relationship:

(4) $$\text{OCR=}{\sigma^{\prime }}_{\text{p}}/\sigma^{\prime }$$

The incremental vertical strain is calculated for every time step for each layer as a function of effective stress, in this case solely derived from hydraulic head changes. The model only considers vertical deformation. Horizontal displacement is assumed negligible at delta scale (Ye et al 2016). The hydrological effect of viscous (creep) compression, which tends to increase pore pressure and therefore hydraulic head, was set to zero, as the model was calibrated without this budget term.

Parameterization for the abc compression constants was based on local geotechnical data (Bakr et al 2013, Thoang and Giao 2015, Toan and Nu 2013) combined with general relationships among compression parameters known from other studies (see online supplementary S5 for a detailed description and summary of the used values). Deformation behaviour is strongly determined by the overconsolidation ratio, but only limited data is available to constrain the OCRs for the MKD. Hoang et al (2016) determined OCRs ranging between 1.0 and 2.7 (average ∼1.6) for clayey deposits using cone penetration, incremental loading oedometer and constant rate of strain consolidation tests from five boreholes in the MKD. Thoang and Giao (2015) reported an OCR value of 1.6 for medium to stiff clays in HCMC province. OCRs published for comparable delta deposits are within a similar range: Bangkok, Thailand 1.5 (Phien-wej et al 2006); Belfast, UK 1.2–1.8 (Crooks and Graham 1976) and 1.6–2 (Bell 1977 in Graham et al 1983). Based on these values, we established an initial OCR range of 1.2–2. As there is no known trend or consistency on how OCR changes with depth in the MKD (Hoang et al 2016), a single OCR value was assigned to all model layers. A sensitivity analysis of modelled subsidence to OCR was used to further constrain the range of possible OCR values. Subsequently, the OCR of the model results that shows the highest correlation to the InSAR-measured subsidence (Erban et al 2014) for the MKD was determined.

## 3. Results

### 3.1. Modelled aquifer drawdown

**Figure 4.**
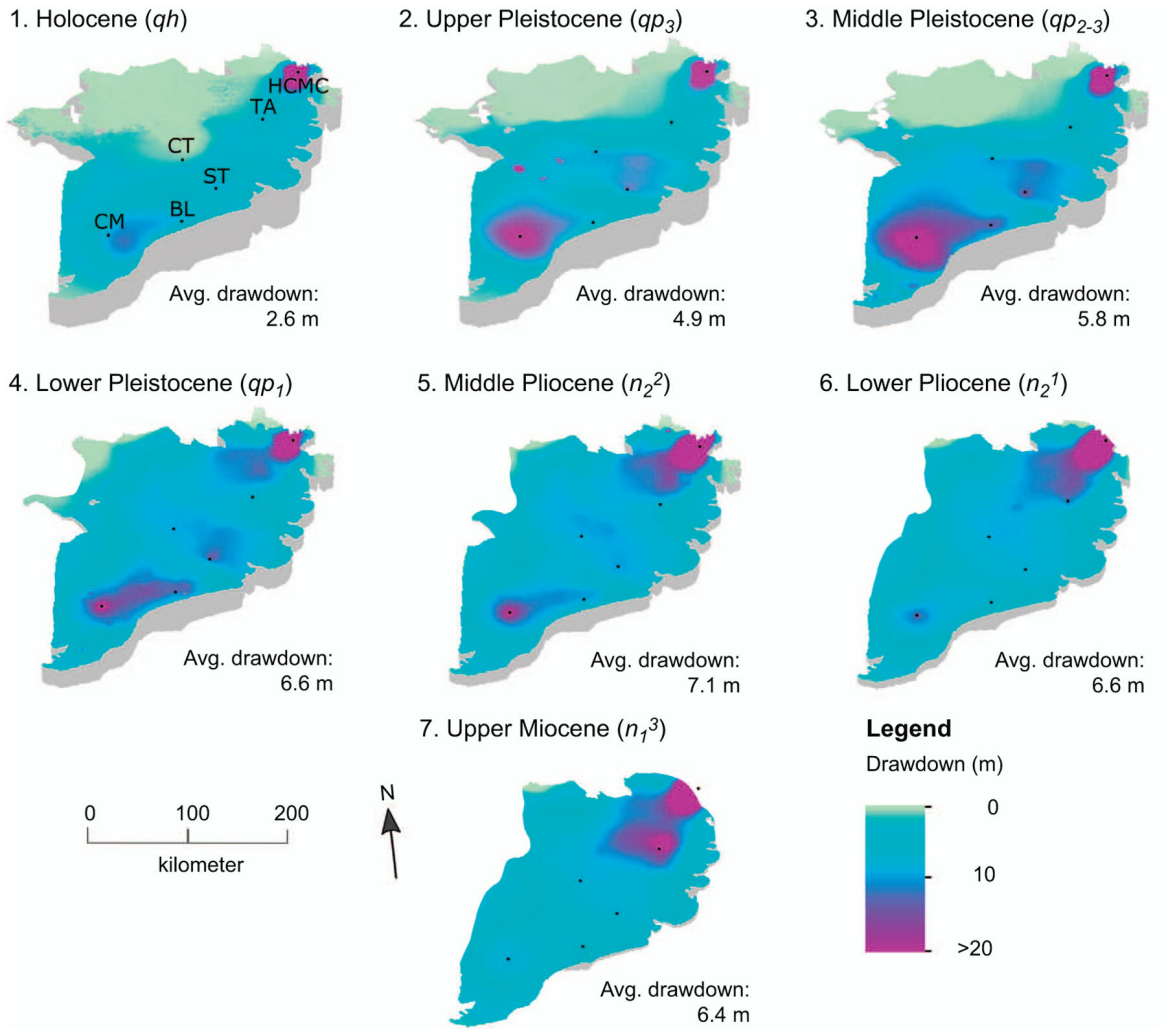
Modelled aquifer drawdown at the start of 2016 after 25 years of simulated groundwater extraction for in the seven main aquifers of the MKD (see figure 2(a)). The deeper aquifers do not extend over the entire delta. Cities located in the figure: BL: Bac Lieu; CM: Ca Mau; CT: Can Tho; HCMC: Ho Chi Minh City; ST: Soc Trang; TA: Tan An.

For the simulated period of 25 years, the calibrated hydrogeological model produces groundwater heads correlating reasonably well with the observed time series of groundwater head in the MKD (r^2^ =0.73). The residuals of the absolute observed versus absolute modelled hydraulic heads are normally distributed with over 75% smaller than 2 m. For HCMC, the residuals are larger and observed drawdown at the observation wells is consistently underestimated several meters by the model (online supplementary S6). Despite such systematic offsets, the decrease of head over time matches well (median cross-correlation (r = 0.94). This provides a good basis for the subsequent subsidence calculations, as they strongly depend on the hydraulic head change, rather than absolute values. Over the past 25 years, large areas in the MKD experienced drawdown in the aquifers exceeding 5 m (figure 4). Modelled average delta-wide drawdown increases with depth and large drawdown areas are located around major cities and industrial areas with extensive groundwater extraction, e.g. Bac Lieu, Ca Mau, Soc Trang and Tan An. HCMC has extensive cones of depression modelled in all aquifers, with local groundwater levels reduced well over 20 m, locally up to 40 m.

### 3.2. Effect of overconsolidation ratio on subsidence modelling

**Figure 5.**
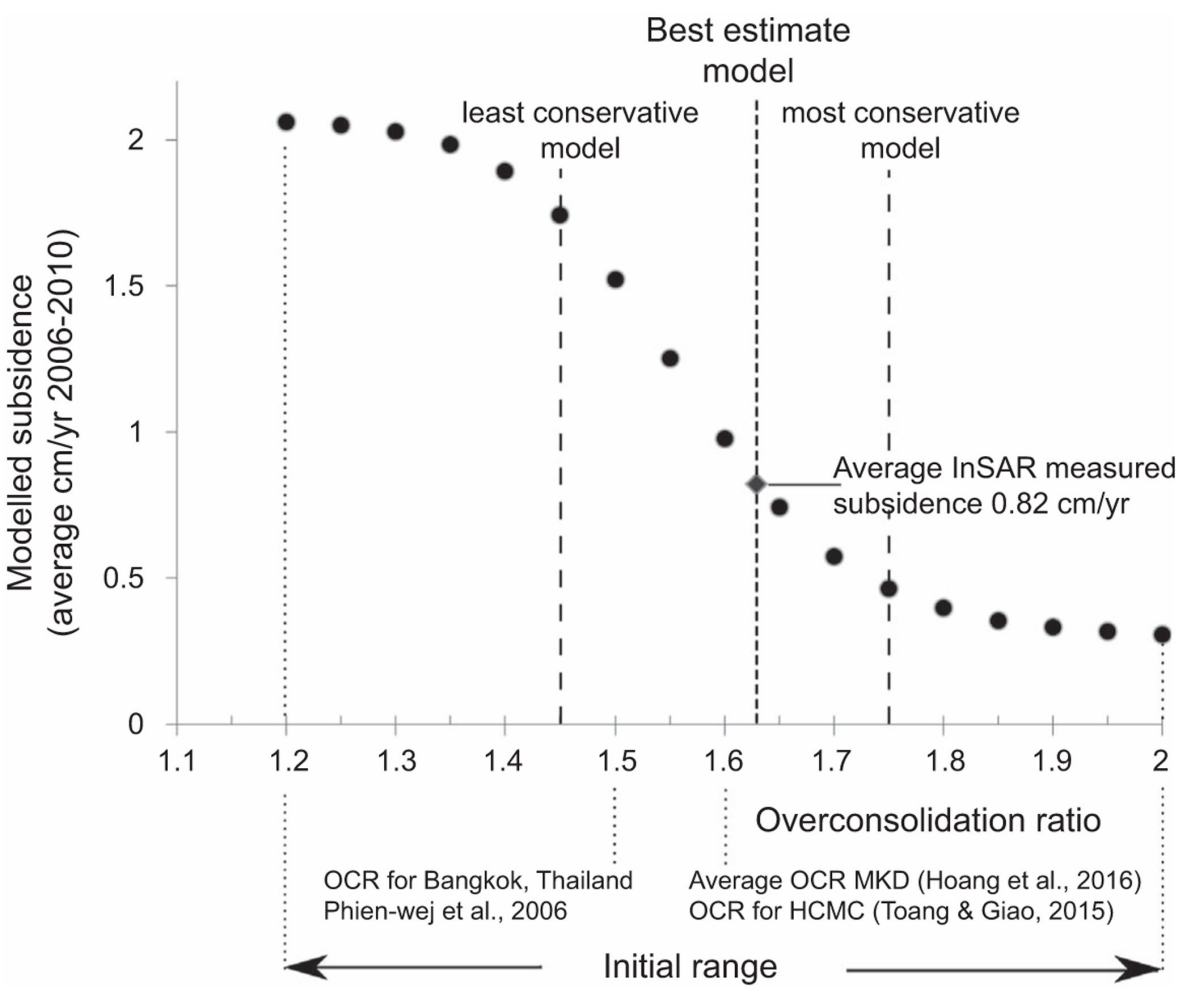
Sensitivity analysis of modelled subsidence to overconsolidation ratio. The range of OCR values of 1.45–1.75 represents the determined least to most conservative subsidence model. The model using an OCR of 1.63 has the highest correlation with the average InSAR-measured subsidence for the entire MKD (calculated from the Erban et al 2014) and is selected as the best estimate model. Rates are all average annual value over the period 2006–2010.

Subsidence modelling of aquifer-system compaction related to hydraulic head drops strongly depends on the overconsolidation ratio attributed to the model. By scrutinising the sensitivity analysis of modelled subsidence to the initial OCR range, the plausible OCR range could be decreased (figure 5). Low OCR values (<1.45) result in an unrealistic rapid subsidence response, i.e. very high viscous creep rates, even without groundwater extraction. High OCR values (>1.75) result in very limited to zero viscous response of the aquifer system, which is very unlikely in a delta system. The 1.45–1.75 OCR-range provides a series of subsidence calculations from a least conservative (very weak sediments) to a most conservative model (rigid sediment properties). If no direct measurements of subsidence are available for a delta system, this would be the range in which groundwater extraction related subsidence can be reported. In case of the MKD, we determine the best estimate model by correlating the average model results to the average InSAR-measured subsidence rates for the entire MKD for the period 2006–2010 (Erban et al 2014). The model parameterized with an OCR of 1.63 provides the best match with the average InSAR-measured subsidence and was consequently used to calculate the reported results. This OCR value approaches the reported average OCR value of 1.6 reported for the MKD (Hoang et al 2016) and HCMC (Thoang and Giao 2015). The amount of the total InSAR-measured subsidence reproduced by the model, resulting from a cell-by-cell comparison, was ∼75% of the total measured subsidence for the best estimate model (respectively ∼50% and ∼95% for the most and least conservative model) (online supplementary S7).

### 3.3. Groundwater extraction-induced subsidence in the MKD

**Figure 6.**
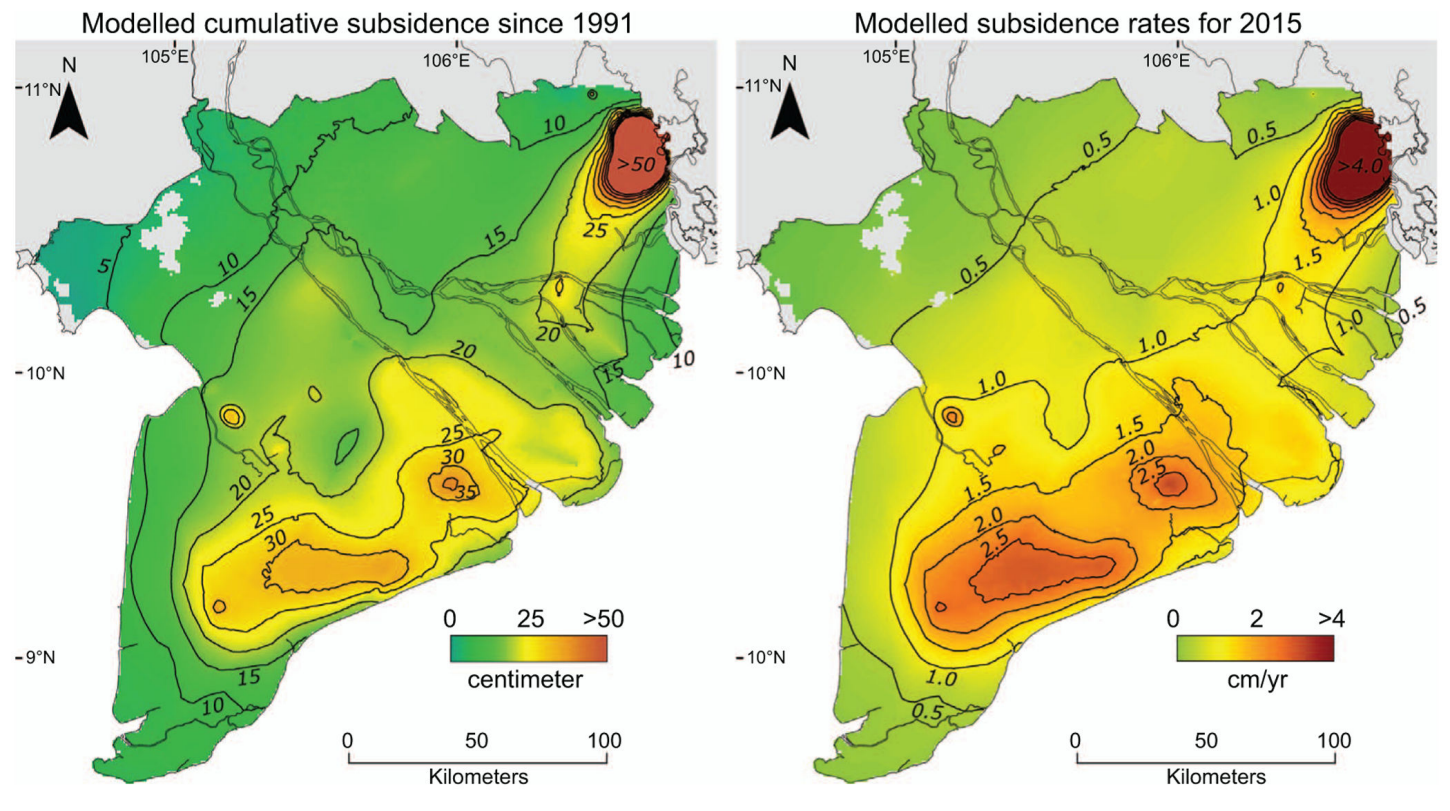
(*a*) Modelled cumulative subsidence due to groundwater extraction-induced during 25 years of groundwater exploitation from 1991 to 2016. (*b*) Modelled groundwater extraction-induced annual subsidence rates for 2015.

The model indicates that since 1991, 25 years of groundwater exploitation in the MKD has resulted in an average total subsidence of ∼18 (9–53) cm for the best estimate (most conservative and least conservative) model, respectively, with hotspots over 30 (18–75) cm (figure 6(a) and online supplementary movie). Cumulative subsidence values calculated for HCMC well exceed those figures, on average ∼115 (*90–150*) cm). The modelled average sinking rate for 2015, solely due to 25 years of groundwater extraction, is 1.1 (*0.7–1.8*) cm yr^−1^. Cities and major industrial areas particularly stand out with high subsidence rates (up to 2.5 (*1.7–3.3*) cm yr^−1^) while rates for rural areas with substantial groundwater extraction generally range from 1–2 (*0.6–3.1*) cm yr^−1^ (figure 6(b)). Present average modelled subsidence rate for HCMC is ∼7.3 (*6.6–7.7*) cm yr^−1^.

**Figure 7.**
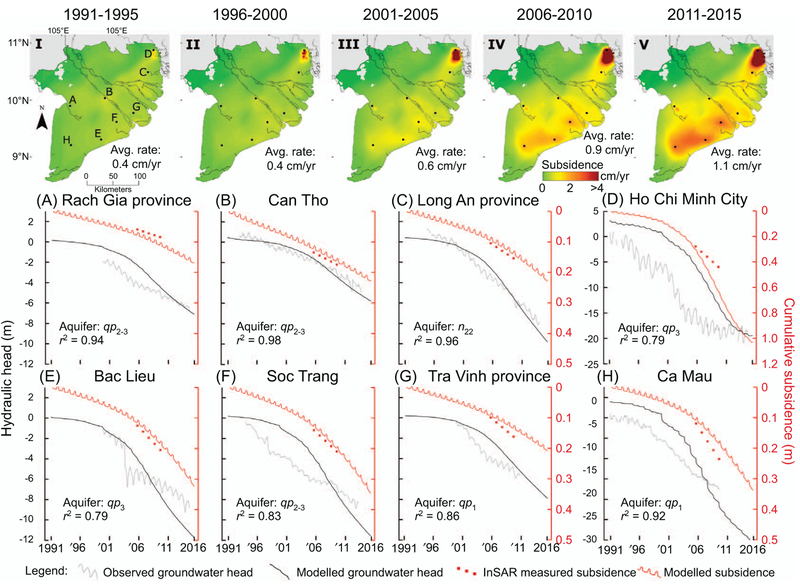
(i)-(v) Annual groundwater extraction-induced subsidence rates for each five year period. Monitoring well locations are marked alphabetically. (*a*)-(*h*) Modelled and measured hydraulic head time series at monitoring well locations. Cumulative calculated subsidence is shown in red. The periodic fluctuations in the subsidence graphs reflect the elastic response up to 2 cm to seasonal wetting and drying as the aquifer system expands and shrinks. The red dots represent InSAR-measured subsidence rates over 2006–2010 by Erban *et al* (2014) for visual comparison.

At the start of the modelling period in 1991, the hydrogeological situation for the vast majority of the MKD was in a natural, undisturbed state. With the exception of Ca Mau city, hydraulic heads were at surface elevation and locally artesian. Groundwater extraction started to exceed aquifer recharge at many locations between 1991 and 1995, initiating widespread hydraulic head decline. As extraction rates continued to increase (figure 3), hydraulic head declines accelerated throughout the multi-aquifer system (figure 7(a)–(h)). The modelled heads follow the measured hydraulic heads in this period, although during the middle part of the modelling period, the modelled head decline is lagging behind the observed head decline. Substantial subsidence commenced during the 1990s as a result of groundwater pumping-induced aquifer compaction in large parts of the delta (figure 7(i)–(v). Annual rates steadily increased towards the present as aquifer depletion persisted. For the MKD, the highest subsidence rates are found at the end of the modelling period. In HCMC a slight decrease in subsidence rate is visible towards the present, following the recent decrease in aquifer depletion (figure 7(d)).

## 4. Discussion

### 4.1. Contribution of groundwater extraction to total subsidence in the mekong delta

Our model calculates subsidence resulting fromaquifer-system deformation when subjected to groundwater over-extraction. In general, subsidence includes contributions by other drivers (e.g. Tosi et al 2009). Apart from groundwater extraction, these include (e.g. Galloway et al 2016): 1) shallow subsidence in the unsaturated zone triggered by phreatic groundwater level lowering, 2) natural and anthropogenic loading by for example buildings and infrastructure and 3) deeper-rooted tectonics. For the MKD, in ∼75% of the cases the InSAR-measured subsidence is at least matched by the best estimated modelled subsidence, respectively ∼50% to ∼95% for the most conservative to the least conservative model (online supplementary S7). These numbers provide an estimate of how much of the InSAR-measured subsidence might be caused by groundwater extraction. Even though groundwater extraction seems to explain a large part of the measured subsidence, a large part of the InSAR-measured subsidence is unaccounted for by the model results. In addition, InSAR measurements may underestimate total subsidence in a delta, as InSAR measurements are relative measurements within individual satellite imaging swaths (∼50–100 km) and can miss additional regional-scale subsidence unless calibrated with ground-based GPS.

Locally, underestimation by InSAR of total subsidence may occur where the InSAR signal was reflected from objects (e.g. buildings) founded on deeper sediment layers, and thus not register any shallow subsidence occurring between the foundation and the delta surface. Groundwater extraction is but one component of the entire InSAR-measured subsidence signal, which may include other factors such as young sediment consolidation or motion along faults. Other subsidence drivers are likely contributing to the total subsidence in the MKD and to the InSAR measurements of surface deformation.

The estimates of groundwater extraction-induced subsidence resulting from this studysuggest ground-water extraction as a dominant driver of subsidence in the MKD, supporting previous indications (Erban et al 2013, 2014). This is in line with observations from other subsiding deltas, such as the Yellow river delta (Higgins et al 2013) and delta cities e.g. Bangkok (Chao Phraya delta, Phien-wej et al 2006), Suzhou (Yangtze delta, Shi et al 2012), Jakarta (Abidin et al 2011), numerous Indonesian cities (Chaussard et al 2013), Shanghai (Wu et al 2010, Ye et al 2016) and many more (e.g. Holzer and Johnson 1985, Gambolati and Teatini 2015). Where the above-mentioned studies often focused on a single city or a relatively small part of a delta, our work now provides quantitative estimates for nearly the entire delta system, including all its cities and rural areas, and demonstrates spatial differences in subsidence due to groundwater extraction.

Overall, groundwater extraction-induced subsidence in the MKD seems to be highest in urban and industrial areas, where high, concentrated groundwater usage creates local subsidence hotspots. In the rural parts of the delta subsidence rates are slightly lower. Still, as most of the delta comprises rural areas, the collective extractions by millions of local people for domestic and agricultural use are responsible for ∼80% of the total extracted volume in the MKD (DWRPIS 2010), and therefore they are the largest contributor to groundwater extraction-induced subsidence at the delta scale.

### 4.2. Robustness of modelling results

**Figure 8.**
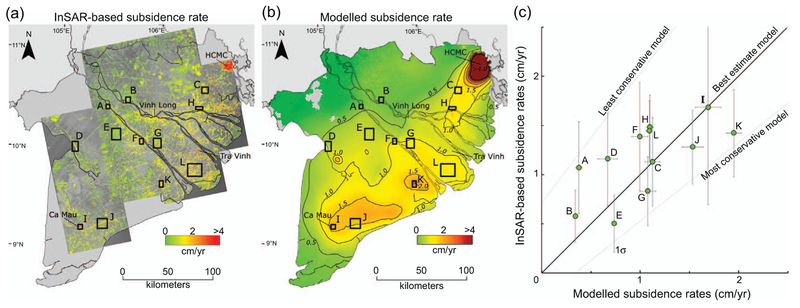
(*a*) InSAR-measured subsidence (after Erban et al 2014, data © JAXA, METI 2011). Rectangles show selected subsets for comparison. A: Long Xuyen; B: Cao Lahn C: Tan An; D: Rach Gia; E: Can Tho province; F: Can Tho; G: Vinh Long province; H: My Tho; I: Ca Mau; J: Bac Lieu province; K: Soc Trang; L: Tra Vinh province. (b) Modelled subsidence of the best estimate model (OCR:1.63). (c) Fit between modelled subsidence rates and InSAR measurements for the selected subsets showing average values (green dots) with one standard deviation (σ). The linear fit trend line (y = 1.0x) of the best estimate model and respectively the least and most conservative models are shown (data points are not shown). All rates are in annual averages over the period 2006–2010.

Our best estimate model produces a subsidence pattern for the MKD that shows similarities to the subsidence portrayed by the InSAR measurements, with a large, subsiding region extending from HCMC (in the northeast) to Ca Mau province (in the southwest)(figure 8(*a*)–(*b*)). For a more detailed comparison, we selected twelve subsets in the MKD with 1) a clear InSAR signal and 2) substantial groundwater extraction. The areas cover major cities as well as rural parts of the delta. Almost all average measured and modelled subsidence values within the comparison windows fall within 0.5 cm yr^−1^ of the linear fit for the best estimate model (figure 8(c)). This value is similar to the average uncertainty range reported for the InSAR derived subsidence rates (Erban et al 2013). Furthermore, all average subset values fall within the a priori defined least to most conservative model range, underscoring the potential to determine an acceptable subsidence model parameterization range (i.e. OCR) even when validation data is lacking. The least to most conservative models produce annual subsidence rates of respectively ∼160% and ∼60% of the best estimate model. As such, this range is taken as uncertainty range for the reported modelling results.

### 4.3. Subsidence in the MKD

At several locations in the delta the InSAR-measured subsidence rates do not match well with the modelled subsidence. For example, modelled subsidence is appreciably lower for the cities of Tra Vinh and Vinh Long and the rural area south of HCMC. In addition to the aforementioned uncertainties associated with each method, we have three possible explanations. Firstly, the observed subsidence is largely caused by shallow subsidence, unrelated to groundwater extraction and therefore unaccounted for by the model. This effect may be especially important in coastal areas with young, recently deposited, superficial sediments with high consolidation potential. Surface elevation table (SET) measurements in three coastal mangrove areas in the MKD indeed reveal fairly high near-surface consolidation rates, ranging from 1.4–4.1 cm yr^−1^, (Giao et al 2014, Lovelock et al 2015). Secondly, as subsidence rates can vary over relatively short distances associated with differences in subsurface conditions, as has been observed in the Rhine–Meuse delta (Asselen et al 2009, Koster et al 2016), the Mississippi delta (Törnqvist et al 2008) and the Ganges–Brahmaputra delta (Higgins et al 2014), the model is unable to reproduce local subsidence resulting from subsurface heterogeneity beyond the resolution of the subsurface discretization but captured by high-resolution InSAR measurements. Thirdly, groundwater extraction in the model is based on officially registered and estimated extractions by the DWRPIS, which is at present the best available source of data on groundwater use. However, uncertainty and/or deficits of groundwater extraction in the records, for example due to unregistered extractions, likely influence model results locally (discussed further in online supplementary S3.3).

### 4.4. Subsidence in HCMC

Modelled subsidence rates for HCMC exceed the InSAR-measured subsidence (Erban et al 2014) in both spatial extent and magnitude. A detailed InSAR analysis of HCMC by Minh et al (2015) shows annual subsidence rates up to 7 cm, matching the average modelling results for the city, but spatial patterns differ. Minh et al (2015) attribute spatial subsidence variations in HCMC to subsurface heterogeneity that cannot be captured by the 3D spatial resolution of our current delta-wide subsurface model. Additionally, at several locations in the city with high extraction well density, modelled drawdown rates greatly exceed head declines monitored at the fringes of the city. This leads to questionable annual subsidence rates go up to several decimetres. Possible explanations for overestimated drawdown include underestimation of local aquifer connectivity and the absence of the Saigon river system (not modelled), resulting in lower recharge values. For this reason, we reported only average simulated subsidence values for HCMC, as the current model and available data does not permit more detail.

Nonetheless, all estimates suggest the HCMC law to limit groundwater use (HCMC 2007) seems to have had an effect. Extraction figures have stabilised since 2007 and decelerating drawdown rates over the past years are both measured and modelled. Consequently, the associated subsidence also shows a slight deceleration towards the present (figure 7(d)).However, as subsidence is a slow responsive process and with hydraulic heads still well below initial levels, subsidence is on-going.

### 4.5 Future outlook for the MKD

As the MKD continues to develop and industrialise, groundwater exploitation is likely to increase further in the decades to come. In rural areas, conversion of land use practice to more groundwater-intensive businesses, e.g. from two to three rice crops, or paddy to shrimp ponds, is on-going (e.g. Renaud et al 2015). Our modelling results indicate that pumping-induced subsidence rates in the MKD continuously increased over the past 25 years, with present rates (delta-wide average of 11 (*7–18*) mm/yr, with areas surpassing 25 (*17–33*) mm/yr) exceeding local rates of absolute sea level rise by an order of magnitude (∼3 mm yr^−1^; Church et al 2013). These rates are alarming given that the majority of the MKD land surface is less than 2 m above mean sea level, while subsidence rates may increase further.

Elevation loss resulting from subsidence increases flood and storm surge vulnerability. The MKD and its cities are likely to experience more frequent and prolonged inundation periods. This trend is already apparent in the cities of Can Tho (Huong and Pathirana 2013, Takagi et al 2016) and HCMC (Phi 2008). Moreover, subsidence increases salt water intrusion in the estuaries and the delta’s dense network of surface waterways, in turn increasing the pressure on groundwater reserves.

## 5. Conclusions

Our process-based approach, employing the first delta-wide, one-way coupled 3D hydrogeological and subsidence numerical model of the MKD, enabled us to compare groundwater extraction-related subsidence to total InSAR-measured subsidence at delta scale. This is an important step towards disentangling the total, measured subsidence signal for a delta into different drivers of subsidence. The approach also facilitates the analysis of the timing of hydraulic head decline and corresponding subsidence during the modelling period. When sufficient hydrological and geological data is available, this modelling approach can be applied to other delta systems worldwide facing groundwater-extraction related subsidence, to estimate a range of subsidence rates even when no direct subsidence measurements, such as InSAR, are available. When available, direct measurements of subsidence form valuable datasets to confine the range of groundwater extraction-induced subsidence estimates.

In case of the Vietnamese MKD, the hydro-geological system of the MKD has been transformed from an almost undisturbed to a human-impacted state with accelerating aquifer depletion due to increasing groundwater extraction during the past 25 years. Aquifer system compaction following dropping water pressures in the aquifers has resulted in dramatic delta subsidence over this period.

Our best estimate model suggests that a quarter-century of pumping-induced subsidence caused the MKD to sink on average by ∼18 cm over the past 25 years, with areas over 30 cm. At present, the average groundwater extraction related subsidence rate in the MKD lies around 1.1 cm yr^−1^, with local extremes over 2.5 cm yr^−1^. For HCMC current rates are as high as ∼7 cm yr^−1^. Groundwater extraction seems to be a major subsidence driver in the MKD, as indicated by both our model and previous InSAR-measured subsidence. However, other drivers likely contribute substantially to the total subsidence experienced in the delta as well.

The alarming subsidence rates reported in this study showcase the real and urgent threat groundwater extraction related subsidence can pose to low-lying deltas like the MKD, exacerbating flood vulnerability, saltwater intrusion and coastal erosion. For this reason, delta subsidence should be a priority for responsible policy makers, effective policy strategies could curtail subsidence caused by groundwater extraction. In Vietnam, this is already demonstrated for HCMC where the restriction on groundwater overexploitation seems to alleviate subsidence. Monitoring subsidence by measuring total surface elevation change, e.g. by InSAR, LiDAR or GPS, and in-situ, depth-dependent subsidence, e.g. by SETs, extensometers or benchmarks is essential to facilitate management decisions in subsiding deltas and should be invested in. In addition, 3D numerical models, as presented in this study, have the potential to provide highly relevant predictions of delta-wide subsidence, supporting the urgently needed decision-making in subsiding deltas.

## Supplementary Material

Sup 2

Supplement1

## Figures and Tables

**Figure 2.**
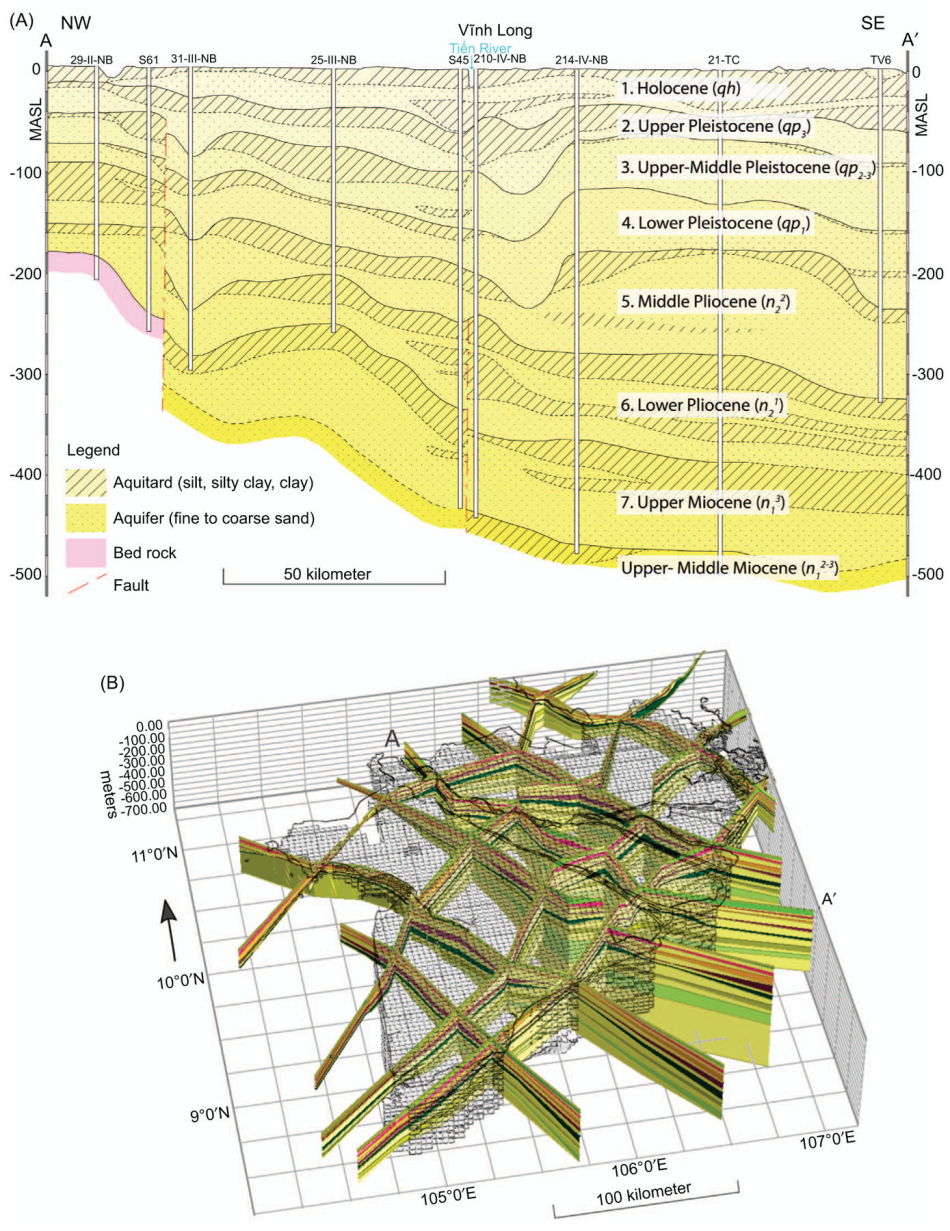
(*a*) Hydrogeological cross-section with the interpretation of the deltas subsurface aquifer-system identifying the main units according to the Division of Geological Mapping for the South of Vietnam (modified after DGMS 2004). Each unit consists of a permeable bottom layer (aquifer) and an, occasionally discontinuous, confining top layer (aquitard). (*b*) Ten hydrogeological cross-sections distinguishing aquifers and aquitards used to create the 3D subsurface model, by linear interpolation, of the MKD. The cross-sections are linearly extrapolated into the sea and cross-border to reach the model boundary 50 km offshore and 20 km outside the national border.
