# Supplementary material for: Impacts of 25 years of groundwater extraction on subsidence in the Mekong delta, Vietnam
Source: Environ Res Lett. Author manuscript; Available in PMC 2018 Oct 17. (PMC6192430; doi:10.1088/1748-9326/aa7146)
Supplement: Supplement1 [file NIHMS1503207-supplement-Supplement1.pdf]

Supplementary Information for

Impacts of 25 years of groundwater extraction on subsidence in the Mekong delta, Vietnam

P.S.J. Minderhoud[1,2], G. Erkens[2,1], V.H. Pham[1,2,3], V.T. Bui[3], L. Erban[4], H. Kooi[2], E. Stouthamer[1]

[1]Dept of Physical Geography, Utrecht University, Utrecht, the Netherlands
[2]Department of Subsurface and Groundwater Systems, Deltares Research Institute, Utrecht, the Netherlands
[3]Division of Water Resources Planning and Investigation for the South of Vietnam (DWRPIS), Ho Chi Minh City, Vietnam
[4]US EPA Office of Research and Development, National Health and Environmental Effects Research Laboratory, Atlantic Ecology Division, Narragansett, RI, USA

The following document provides additional description and presentation of data and methods as referred to in the manuscript.

S1. Subsurface model

We adopt the geological subsurface schematization of the Mekong delta (MKD) and the Saigon delta made by the Division for Geological Mapping for the South of Vietnam (DGMS, 2004). As these two delta systems share the same depositional basin, their deposits and groundwater systems are inter-connected. Ten hydrogeological cross-sections based on 95 linearly interpolated, geological borehole logs were used to construct the 3D subsurface model of MKD using a linear interpolation of iMOD SolidTool (Vermeulen *et al* 2016). The deepest confined aquifer (Upper-Middle Miocene age) was not represented in the model due to data paucity. This aquifer is less relevant for this study, as no groundwater extraction is reported for it. Surface elevation was based on a detailed digital elevation model of the Mekong delta supplemented with Shuttle Radar Topography Mission (SRTM) elevation data for areas outside the delta. This resulted in a 15 layered, subsurface model, representing 7 aquifers, 7 aquitards and a phreatic top layer (Table S1). The minimum layer thickness was set to two meters, as required for numerical functioning of the current build of the subsidence module in iMOD (SUB-CR). Furthermore, an adjustment was made in Soc Trang province: an incorrectly interpreted aquitard thickness in a geological borehole log was manually corrected to a minimum thickness of 5 meters. In the current subsurface model, ~60% of the total volume is represented as aquitard (clay and silt) and ~40% as aquifer (sand). A lithological analysis of over 700 core logs revealed a sand versus silt and clay presence for the Mekong delta of respectively 60% and 40%. This implies that about 1/3 of the aquitards in the current subsurface model actually consist of sand, which is reflected by the abundant sand lenses present in the core logs. This is taken into account in the subsidence calculations to avoid overestimation of subsidence.

Table S1. Geological and hydrogeological units of the MKD and corresponding model discretization. In total ~40% of the subsurface presented in the model is aquifer and ~60% is aquitard. Model layer 1 is not shown in this table, as it is not based on a specific geological formation, but represents the phreatic top layer at the delta surface.

**Age**	**Subseries**	**Geological unit**	**Hydrogeological unit**	***Model Layer***	***Hydrogeological unit***	***Average depth below surface (m)***	***Average layer thickness (m)***
**Holocene**	Upper	Q_{II}^{3}	qh_3	2	Aquitard 1	9	18
	Middle/Lower	Q_{II}^{2-3} / Q_{II}^{1}	qh_{2-3} / qh_1	3	Aquifer 1	29	24
**Pleistocene**	Upper	Q_{I}^{3}		4	Aquitard 2	53	22
			qp_3	5	Aquifer 2	70	14
	Middle	Q_{I}^{2-3}		6	Aquitard 3	97	39
			qp_{2-3}	7	Aquifer 3	123	13
	Lower	Q_{I}^{1}		8	Aquitard 4	149	39
			qp_1	9	Aquifer 4	180	21
**Pliocene**	Upper/Middle	N_2^{3}/ N_2^{2}		10	Aquitard 5	212	44
			n_2^{2}	11	Aquifer 5	242	18
	Lower	N_2^{1}		12	Aquitard 6	271	38
			n_2^{1}	13	Aquifer 6	302	21
**Miocene**	Upper	N_1^{3}		14	Aquitard 7	332	41
			n_1^{3}	15	Aquifer 7	382	59

S2. Hydrogeological model setup Table S2. Description of the hydrogeological model setup

**Simulation setup**	**Description**
Boundary conditions	
Recharge	Measured precipitation (1991-2010); average values used (20 11-2015)
Evaporation	Measured evaporation (1999-2010); average values used (1991-1998/20 11-2015)
Drainage	-0.5m below surface elevation
Constant head boundary (sea)	Boundary is set 50 km off-shore with a constant head for layer 1 and 2 equal to mean sea level
No flow boundary (bedrock)	Boundary set at bedrock outcrops at the surface and 20 km across the Cambodian border
Initial conditions	
Hydraulic head	Steady-state simulation without extraction
Start transient simulation	January 15th, 1991
End transient simulation	December 15th, 2015
Solution	
Absolute convergence criteria	0.01
Maximum iteration	15
Time step	30 days (sensitivity to time step discretization was tested and found negligible)
Groundwater extraction	
Integrated database of groundwater extractions	Registered daily amount starting at year of registration combined with estimated of household extractions (see section S3)

S3. Extraction wells

S3.1 Mekong delta

For the MKD two data sets of past groundwater are used, reporting well location (x- and y-coordinates), exploited aquifer and daily extracted volume (DWRPIS, 2010). The first dataset reports on large, industrial extraction wells with an extraction permit (>200m^3/day) covering the start of the modelling period until 2011. The year of registration is assumed to be the starting point of the extraction, and the permitted volume to be the actual daily extraction value. The second dataset is based on a large delta-wide survey held in 2010 using household density and interviews to estimate well depth and daily extraction by the Division of Water Resources Planning and Investigation of South Vietnam (DWRPIS). The outcomes of the survey were grouped in fictive well locations randomly distributed over freshwater zones for each province with extraction rates to match total reported provincial extractions for each aquifer (Table S3). We assumed that the unregistered household groundwater equally grew with the industrial demand. Therefore, the growth recorded in the registered industrial extractions was used to extrapolate the 2010 survey results back to 1991. Following 2011, an annual increase of 2.5% was assumed. This results in a total extraction in the MKD approaching 2.5 million m^3/day at the end of the modelling period (Fig. 3, main article). The Lower Pleistocene (48% of the total volume) and the Lower Pliocene (26% of the total volume) aquifers are the most heavily-exploited in the MKD (Figure S1).

S3.2 Ho Chi Minh province

Three datasets were combined to determine the extraction of groundwater in the HCMC province. The first dataset reports small extractions (<10 m^3/day) per aquifer but lacked location information. The second dataset documented larger extractions (>10 m^3/day) per aquifer including well coordinates. Subsequently, assuming similar extraction patterns, these well locations were adopted for the first dataset (Fig. S2). The year of commissioning for both datasets was unknown. The third dataset discloses registered extractions in the Holocene and Lower Pleistocene aquifer including location and year of construction until 2015. Combined extraction reported by the three datasets (until 2008) nearly matches the total groundwater exploitation reported for 2008 by Thoang and Giao (2015) and Trung *et al* (2010) in Minh *et al* (2015) (Fig. 3, main text). For this reason, the reported extractions were attributed to 2015 and subsequently extrapolated over the modelling period, adopting the volumetric growth captured in the MKD wells (1991-2007) and described in the registered extraction for HCMC (2008-2015). Total extraction modelled for HCMC exceeded 800.000 m^3/day the year 2010 (Table S3).

Table S3. Total modelled groundwater extraction volumes from each aquifer per province in 2010 after integration of several datasets. For HCMC the reported values are for 2008.

Aquifer	q_h	qp_3	qp_{2-3}	qp_1	n_{22}	n_{21}	n_{13}	Total m^3/day
An Giang	8911	55,319	37,789	368	34,185	864	0	137,436
Bac Lieu	0	338	174,319	61,838	12,465	0	0	248,961
Ben Tre	6,859	6,817	5341	0	0	14,961	8,100	42,078
Ca Mau	0	160	265,371	49,859	52,570	5,532	0	373,492
Can Tho	0	2,628	146,872	0	41,972	0	100	191,573
Dong Thap	0	242	1,426	0	114,743	0	0	116,411
Hau Giang	0	11,995	43,234	7,328	0	0	0	62,557
Kien Gian	414	34,046	149,032	5,232	6,835	3,500	0	199,060
Long An	0	431	0	11,491	146,745	35,609	3,869	198,146
Soc Trang	1,869	25,306	189,241	17,186	0	0	11,314	244,916
Tien Giang	0	3,541	0	0	17,376	23,536	99,752	144,205
Tra Vinh	4,698	22	220,175	0	0	0	0	224,896
Vinh Long	3,461	42	18,923	0	73,525	21,304	1,669	118,925
**Total MKD**	**26,212**	**140,889**	**1,251,723**	**153,302**	**500,416**	**105,306**	**124,804**	**2,302,654**
HCMC	3,155	25,699	148,733	219,368	386,247	22,935	4,742	810,880

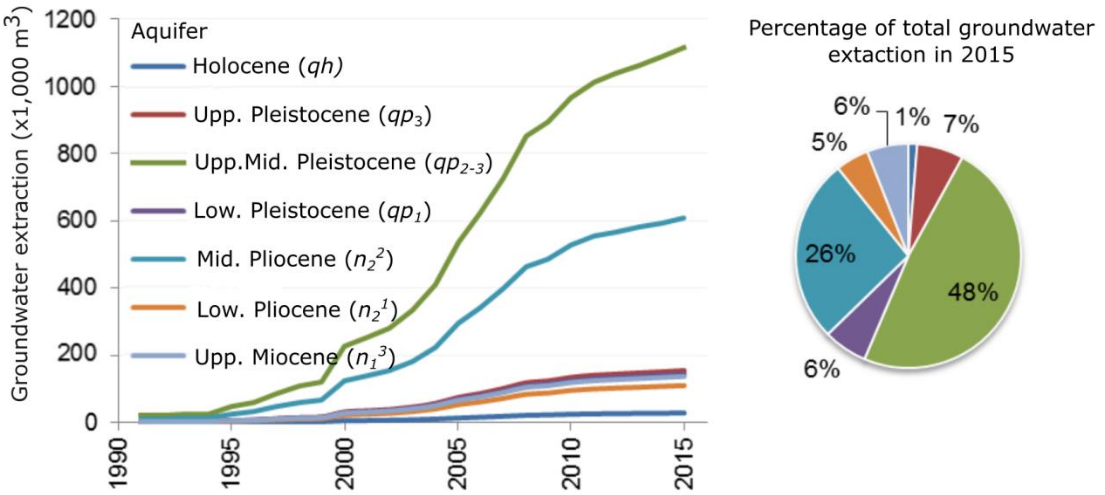

Figure S1. Extracted volume for each aquifer in the MKD. The Middle Pleistocene (qp_{2-3}) aquifer is by far most exploited, followed by the Lower Pliocene aquifer (n_2^2).

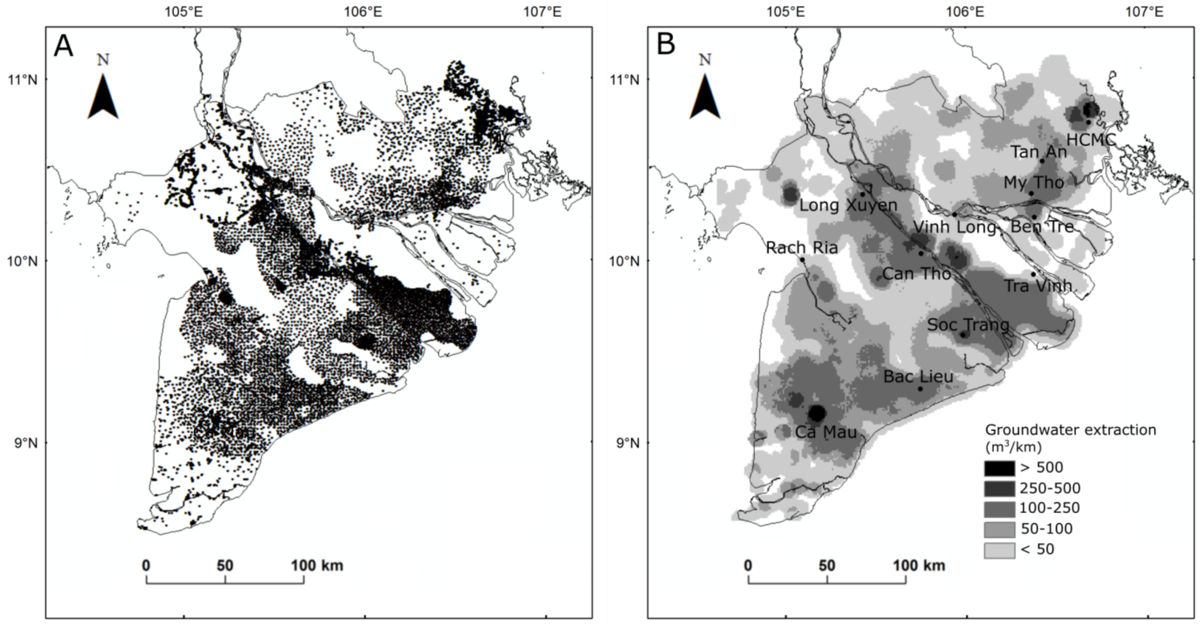

Figure S2. a) Modelled extraction wells and b) extracted volume adopting a 5 km radius around a single well for the year 2015. White areas depict areas with no groundwater extraction reported.

S3.3 Uncertainty in extraction data

We identify four caused of uncertainty in the developed extraction database: 1) The spatial coverage of well data is not all-inclusive (Fig. S2). Several areas where groundwater extraction is suspected are not described in the dataset, e.g. Vinh Long city. 2) Extracted volumes over time are unknown and could be the reason for a possible underestimation of extracted volume during the 90's, resulting in the observed lag of the modelled drawdown with the measured drawdown for that period (Fig. 7, main text). The large increase in documented extracted volume between the year 1999 and 2000 might also be indicative of this underestimation and might be of administrative origin (Fig. 3, main text). For HCMC the mismatch at the start of the modelling period between our dataset and the volumes published by Trung *et al* (2010), in Minh *et al* (2015) potentially indicate a similar underestimation of actual extraction (Fig. 3, main text). 3) Brackish groundwater extraction, e.g. for shrimp farming, is currently not reported while it is known to potentially cause strong drawdowns and consequent subsidence (Higgins *et al* 2013). The absence of brackish groundwater extraction might explain the underestimation of the modelled hydraulic head in saline, coastal areas such as in Ben Tre and Ca Mau province. 4) Seasonal variation in extracted volume is not documented but is likely the case considering the distinct dry and wet season combined with year-round agricultural practices. This could alter the cumulative annual extracted volume, depending on what season the extraction estimates are based, as actual extraction volumes during the dry season are likely higher than during the wet season.

A potential solution for the above-mentioned data deficits is to include extracted volume in the model calibration process, link extraction volume to land use practice and well locations to population density and growth over time. However, adjusting the dataset in this manner would introduce different errors and uncertainties, and requires a lot of detailed local data, as, for example, the main pumping stations producing fresh water for the city of Tra Vinh are located many kilometres away from the city.

S4. Hydrological model parameterization

S4.1 Initial parameterization prior to calibration

Initial values of horizontal hydraulic conductivity (K_h) for the aquifers range from 8.0 to 22.8 m/day and were derived from 999 pumping tests throughout the Mekong and the Saigon delta (DWRPIS, 2010). Aquitard layers were initially parameterized with a K_h value of 0.001 m/day. No measured data was available on the vertical anisotropy of hydraulic conductivity (K_h/K_v). We assumed a vertical anisotropy of 3, as general values of vertical anisotropy for aquifers range between the 1 and 3, with outliers up to 8 (Carlson, 2000). Initial specific storage coefficient (SS_c in m^{-1}) values for individual aquifers (ranging from 1.4 $x10^{-3}$ to 5.6 $x10^{-3}$ m^{-1}) and aquitards (ranging from 3.8$x10^{-5}$ to 2.2 $x10^{-3}$ m^{-1}) are taken from reported values by Haskoning *et al* (1999) and Giao *et al* (2015).

S4.2 Parameter optimisation

The automated parameter estimation in iMOD (i.e. *iPEST,* see Vermeulen *et al* 2016 for a full description of the method) was applied using piezometric head measurements from 91 monitoring wells using ten pilot points (Fig. S3). At every pilot point, K_h and SS_c were systematically adjusted during the PEST procedure (when total model sensitivity to the parameter > 0.5%) and interpolated (simple kriging) over the entire model extend. This was done for each model layer. The maximal multiplication factor allowed for initial values for K_h and SS_c was set to 200 and 1000 for respectively the aquifer and aquitard layers to ensure realistic values. The parameter set creating the smallest error between modelled hydraulic head with measured head time series between 1995-2015 was determined through numerous iterations with each measured time series equally weighted (see also Fig. S5 and S6). Calibrated parameter values are within range of realistic parameter values for the expected sediments (Table S4).

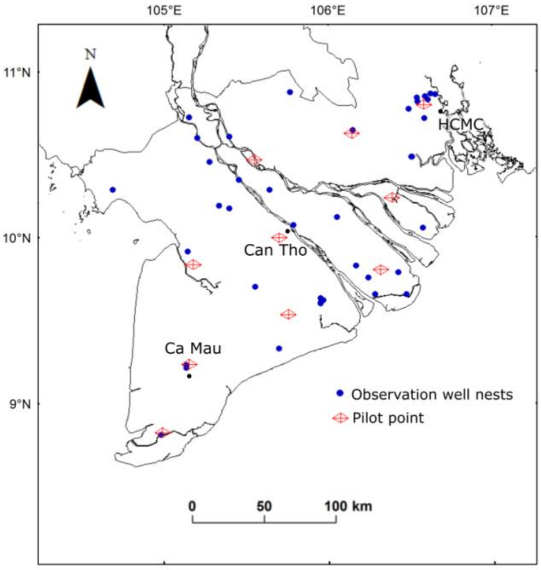

Figure S3 Locations of observation wells and pilot points used during the automated parameter estimation (iPEST).

Table S4 Calibrated values for horizontal hydraulic conductivity and specific storage coefficient following the automated parameter estimation.

Model layer	Description	Calibrated horizontal hydraulic conductivity (K_h in m/day) Median (*mean*)	Calibrated specific storage coefficient (SSc in 1/m) Median (*mean*)
**Layer 1**	Phreatic top layer	5.0 (*5.6*)	3.0x10$^{\wedge-2}$ (*2.9* x10$^{\wedge-2}$)
**Layer 2**	Aquitard 1	3.3x10$^{\wedge-4}$ (*3.2* x10$^{\wedge-2}$)	2.6x10$^{\wedge-3}$ (*4.1 x10*$^{\wedge-3}$)
**Layer 3**	Aquifer 1	8.2 (*6.7)*	1.1x10$^{\wedge-4}$ (*1.4* x10$^{\wedge-4}$)
**Layer 4**	Aquitard 2	1.1 x10$^{\wedge-3}$ (*3.7* x10$^{\wedge-2}$)	3.8x10$^{\wedge-4}$ (*5.0* x10$^{\wedge-4}$)
**Layer 5**	Aquifer 2	46.8 (*499.8*)	1.2x10$^{\wedge-4}$ (*1.9* x10$^{\wedge-4}$)
**Layer 6**	Aquitard 3	180 x10$^{\wedge-2}$ (*3.8* x10$^{\wedge-2}$)	1.6x10$^{\wedge-4}$ (*1.9* x10$^{\wedge-4}$)
**Layer 7**	Aquifer 3	59.5 (*164.4*)	5.8.0x10$^{\wedge-5}$ (*1.0* x10$^{\wedge-4}$)
**Layer 8**	Aquitard 4	5.7 x10$^{\wedge-4}$ (*7.1* x10$^{\wedge-3}$)	1.0x10$^{\wedge-4}$ (*1.6* x10$^{\wedge-4}$)
**Layer 9**	Aquifer 4	11.3 (*10.8*)	5.3x10$^{\wedge-5}$ (*8.4* x10$^{\wedge-5}$)
**Layer 10**	Aquitard 5	3.7 x10$^{\wedge-3}$ (*8.1* x10$^{\wedge-3}$)	7.1x10$^{\wedge-5}$ (*1.7* x10$^{\wedge-4}$)
**Layer 11**	Aquifer 5	67.5 (*148.6*)	5.4x10$^{\wedge-5}$ (1.1 x10$^{\wedge-4}$)
**Layer 12**	Aquitard 6	2.6 x10$^{\wedge-3}$ (*2.9* x10$^{\wedge-3}$)	5.9x10$^{\wedge-5}$ (*2.1* x10$^{\wedge-4}$)
**Layer 13**	Aquifer 6	39.5 (*43.0*)	5.7x10$^{\wedge-5}$ (*1.3* x10$^{\wedge-4}$)
**Layer 14**	Aquitard 7	1.1 x10$^{\wedge-3}$ (*1.1* x10$^{\wedge-3}$)	5.3x10$^{\wedge-5}$ (*2.1* x10$^{\wedge-4}$)
**Layer 15**	Aquifer 7	6.8 (*7.3)*	8.6x10$^{\wedge-5}$ (*5.5* x10$^{\wedge-4}$)

S5. Geotechnical parameterization

S5.1Determining the geotechnical parameters

The geotechnical parameterization for the *abc* model of the MKD subsurface was based on general relationships existing among compression parameters, combined with local geotechnical data. The following section explains the steps taken to determine model parameters. A summary of the used parameters is given in table S5.

As void ratio generally decreases with depth, we empirically derived a depth-dependent void ratio relationship based on a bulk analysis of almost 40,000 geotechnical samples from HCMC province (Bakr *et al* 2013):

$$e_0 = 0.2214 * \ln(z) + 1.5248 \quad (1)$$

Where e_0 is void ratio and z is depth below surface (m). Through this relation, an estimate of e_0 was made for each model layer, using its average depth. The primary compression index (C_c) can be successfully estimated using correlations based on e_0 (Widodo and Ibrahim, 2012). Hence, we determined the C_c for each model layer using the following correlation (Higashi *et al* 2002 in Ohtsubo *et al* 2006):

$$C_c = 0.343 * e_0^{1.328} \quad (2)$$

As sand is on average 20 times less compressible than clays and silts (Table 2.b in NNI, 2012), the C_c for the sandy aquifers was subsequently corrected for this factor.

The recompression index (C_r) was determined using local geotechnical data, following the linear relationship existing between the C_c and C_r indices (Gunduz and Arman, 2007). For the clayey aquitards, we found a C_c/C_r ratio of 5 based on the analysis of Mekong delta clays (Toan and Nu, 2013) and for the sandy aquifers a C_c/C_r ratio of 3, based on data from HCMC (Thoang and Giao, 2015).

Subsequently, the primary compression ratios, CR (compression ratio) and RR (recompression ratio), were derived from the C_c and C_r indices and the void ratio by:

$$CR = \frac{C_c}{1+e_o}, \qquad RR = \frac{C_r}{1+e_o} \quad (3)$$

In principle, for a specific sediment, both compression ratios are independent of depth, however, the above approach introduces a decreasing trend with depth. Physically, this can be explained by a

successive coarsening trend in lithology, which is the case for the Mekong delta (Fig. S4). The derived compression ratios for the shallow aquitards correspond to typical values associated with pure clays, and gradually decrease towards characteristic values for respectively weak sandy clay, sandy clay/loam and sandy loam for the deeper aquitards (Table 2.b in NNI, 2012).

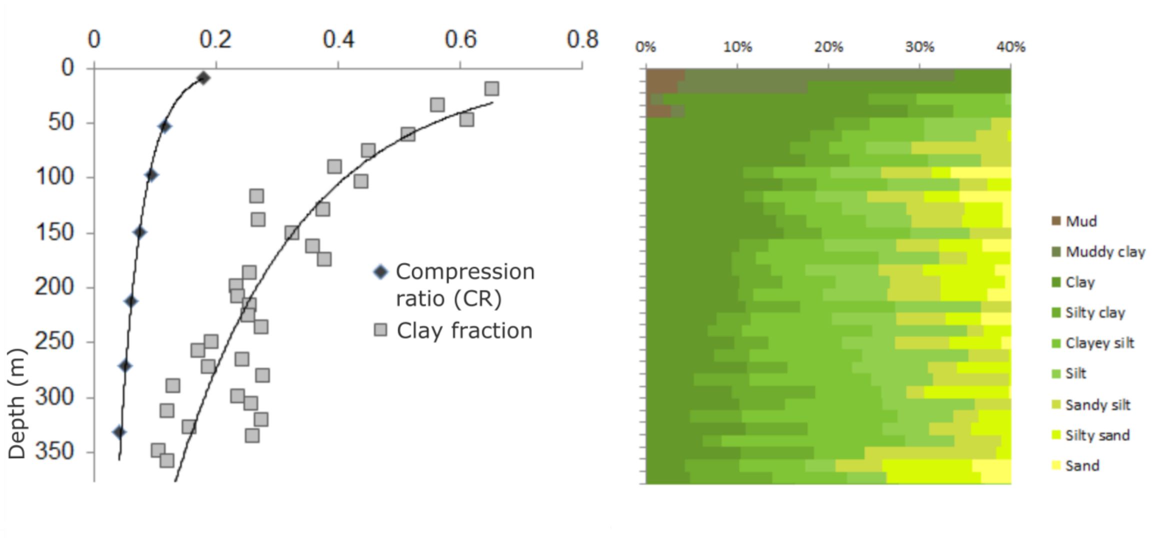

Figure S4 Left: Depth-dependent compression ratio (CR) for aquitards in-line with a decreasing clay fraction with depth. Right: Lithoclass distribution of the 40% finest grained deposits in the Mekong delta, corresponding to the viscoplastic active part of the aquitards in the subsurface model. The coarsening trend in the lithoclasses with depth is clearly visible.

The coefficient of secondary compression (C_α) for the aquitards was determined using a C_α/CR ratio of 0.04 (Mesri and Godlewski, 1977; Ladd *et al* 1977). We assume no secondary compression taking place in the sandy aquifers, therefore C_α is set to zero. For a similar reason, the sandy part in the aquitard layers (1/3 of the total layer thickness) was excluded from compression calculations.

As the *abc* model is based on natural (or Hencky) strain, the final step was to convert the CR, RR and C_α to, respectively, the *a, b* and *c.* For low strain levels, which is the case in the model, the following relationships apply:

$$\begin{aligned} a &= RR / \ln(10) \approx 0.434\,\mathrm{RR} \\ b &= CR / \ln(10) \\ c &= C_\alpha / \ln(10) \end{aligned} \tag{4}$$

Table S5 Parameterization used for subsidence modelling. The top phreatic layer and the bottom aquifer layer in the model were excluded from the subsidence calculations.

**Model layer**	**Description**	***RR***	***CR***	C_α	***a***	**b**	**c**
**Layer 2**	Aquitard 1	3.6E-02	1.8E-01	7.1E-03	1.6E-02	7.8E-02	3.1E-03
**Layer 3**	Aquifer 1	2.3E-03	6.9E-03	0.0E+00	1.0E-03	3.0E-03	0.0E+00
**Layer 4**	Aquitard 2	2.3E-02	1.2E-01	4.7E-03	1.0E-02	5.1E-02	2.0E-03
**Layer 5**	Aquifer 2	1.8E-03	5.3E-03	0.0E+00	7.7E-04	2.3E-03	0.0E+00
**Layer 6**	Aquitard 3	1.9E-02	9.3E-02	3.7E-03	8.1E-03	4.0E-02	1.6E-03
**Layer 7**	Aquifer 3	1.4E-03	4.2E-03	0.0E+00	6.1E-04	1.8E-03	0.0E+00
**Layer 8**	Aquitard 4	1.5E-02	7.6E-02	3.0E-03	6.6E-03	3.3E-02	1.3E-03
**Layer 9**	Aquifer 4	1.1E-03	3.4E-03	0.0E+00	4.9E-04	1.5E-03	0.0E+00
**Layer 10**	Aquitard 5	1.2E-02	6.1E-02	2.4E-03	5.3E-03	2.6E-02	1.1E-03
**Layer 11**	Aquifer 5	9.2E-04	2.8E-03	0.0E+00	4.0E-04	1.2E-03	0.0E+00
**Layer 12**	Aquitard 6	1.0E-02	5.0E-02	2.0E-03	4.4E-03	2.2E-02	8.7E-04
**Layer 13**	Aquifer 6	7.6E-04	2.3E-03	0.0E+00	3.3E-04	9.9E-04	0.0E+00
**Layer 14**	Aquitard 7	8.3E-03	4.1E-02	1.7E-03	3.6E-03	1.8E-02	7.2E-04

S5.2 Implication of the simplified groundwater-subsidence modelling approach

We used a one-way coupling approach to relate hydraulic head development and subsidence in the sense that we first model hydraulic head development with a conventional groundwater model, and then use the hydraulic heads as a function of space and time to drive the subsidence module. However, ideally or formally, a fully (two-way) coupled solution is required to ensure consistency between the strain (or compression) rates in the subsidence module on the one hand, and the storage rates in the groundwater model on the other. The simplified one-way approach was adopted because calibration of the fully-coupled model would demand excessive computation time.

To check the consistency between the independently calibrated specific storage values of the groundwater model and the compression behaviour of the subsidence module, we calculated elastic and 'virgin' specific storage values for the model aquitards from the compression (a or Cr or RR and b or Cc or CR) parameter values and estimates of the effective stress. The calculated elastic values were found to differ from the calibrated values by a factor ranging between 0.05 and 0.4, and the calculated 'virgin' values by a factor ranging between 0.3 and 2.2. The fact that these values are reasonably close to 1 indicates fair consistency. A model run in which the calibrated specific storage values of the aquitards were replaced by the compression-parameter based specific storage values (taking the mean value of the elastic and 'virgin' values) yielded slightly higher subsidence values (average value of cumulative delta-wide subsidence was ~2% higher). Thus although the parameters in the groundwater and subsidence model are not fully coupled, the potential impact on the modelled subsidence values falls well within the reported range of modelled subsidence associated with uncertainty in model parameters in general."

Another uncertainty arises from the current aquitard discretization, which might not be refined enough in a vertical direction to correctly represent the delayed propagation of pressure decline in the less permeable layers. The majority of the model is behaving quasi-steady with very limited delays in pressure decline propagation. This implies that the current layer-discretization is sufficient for this delta-wide model, however it is not the case everywhere in the model and could still contribute to model uncertainty.

S6. Modelled versus measured hydraulic head

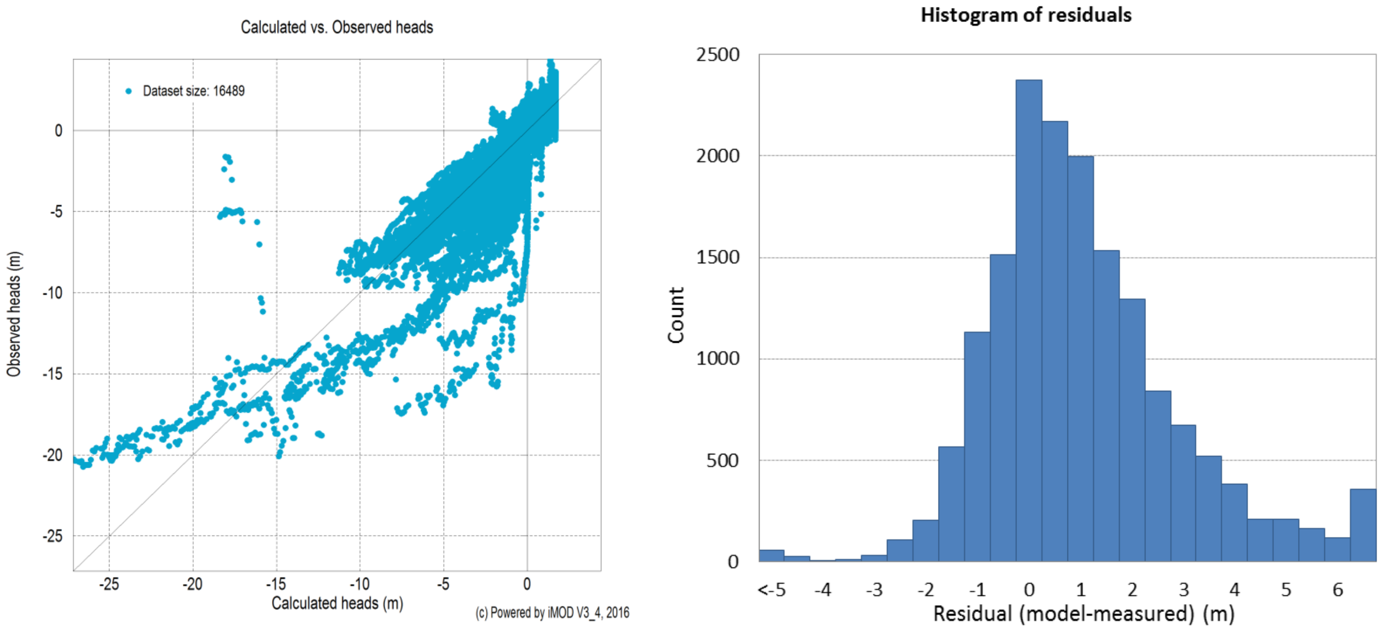

Figure S5 Left: Scatterplot of measured versus modelled hydraulic heads for the MKD. Every point represent a monthly measurement of absolute head versus modelled head for the same month (average r^2 = 0.73; median cross-correlation (r) = 0.94). Right: Histogram of residuals for the MKD. Of the modelled heads, >75% are within 2 meters of observed heads. Largest residuals are from monitoring wells around Ca Mau city. Residuals are positive when modelled head > observed head.

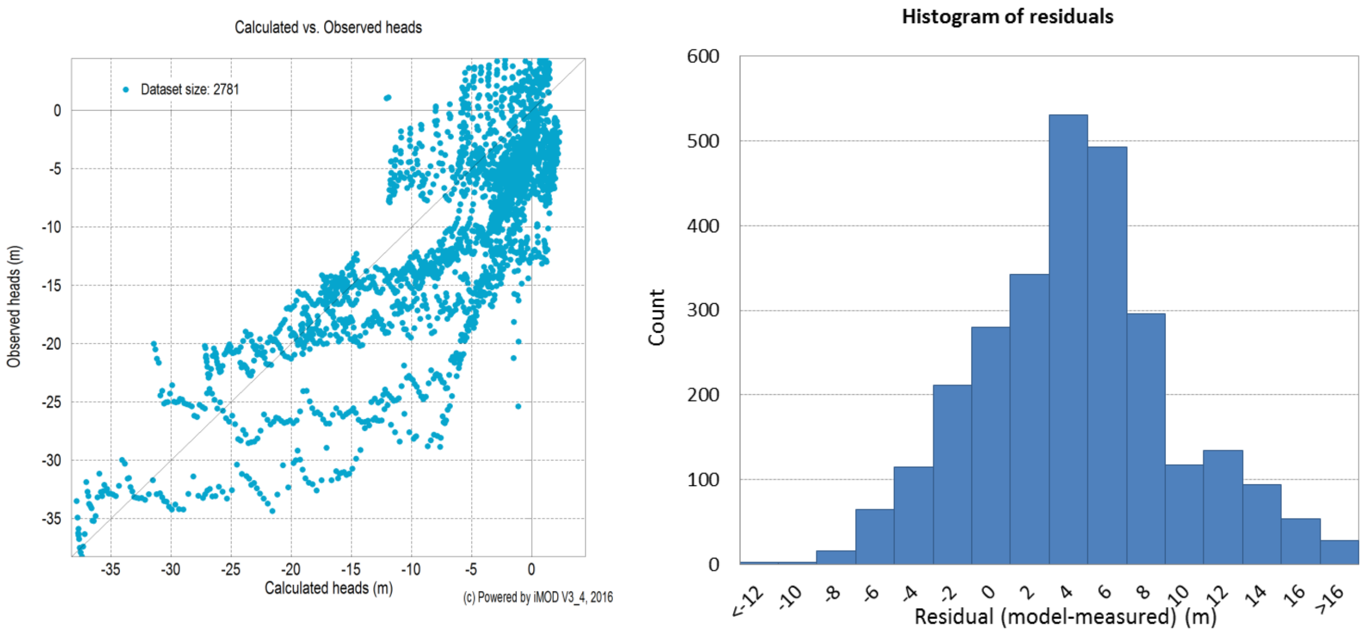

Figure S6 Left: Scatterplot and residual graph of the HCMC wells. Clearly, the observed head increasingly exceeds the calculated head with increased drawdown. Right: Histogram of residuals for HCMC province, showing the structural underestimation of observed heads by the model. Residuals are positive when modelled head > observed head.

S7. Modelled versus measured subsidence

A direct cell-by-cell comparison between upscaled InSAR-measured subsidence (Erban et al., 2014) (see S8 on upscaling) and modelled groundwater extraction-induced subsidence is shown in Fig. S7. The percentages give the part of the total InSAR-measured subsidence reproduced by modelled groundwater extraction-induced subsidence. The calculation is illustrated by the following example for Fig. 9. Point A: 1.5/0.5 cm/yr, measured > modelled subsidence = 33% explained by the model; Point B: 1.5/1.5 cm/yr = measured equals modelled subsidence = 100% explained by the model; Point C: 0.5/1.5 cm/yr, measured < modelled subsidence = 100% explained by the model. The percentages in the figure are the average values of all cells.

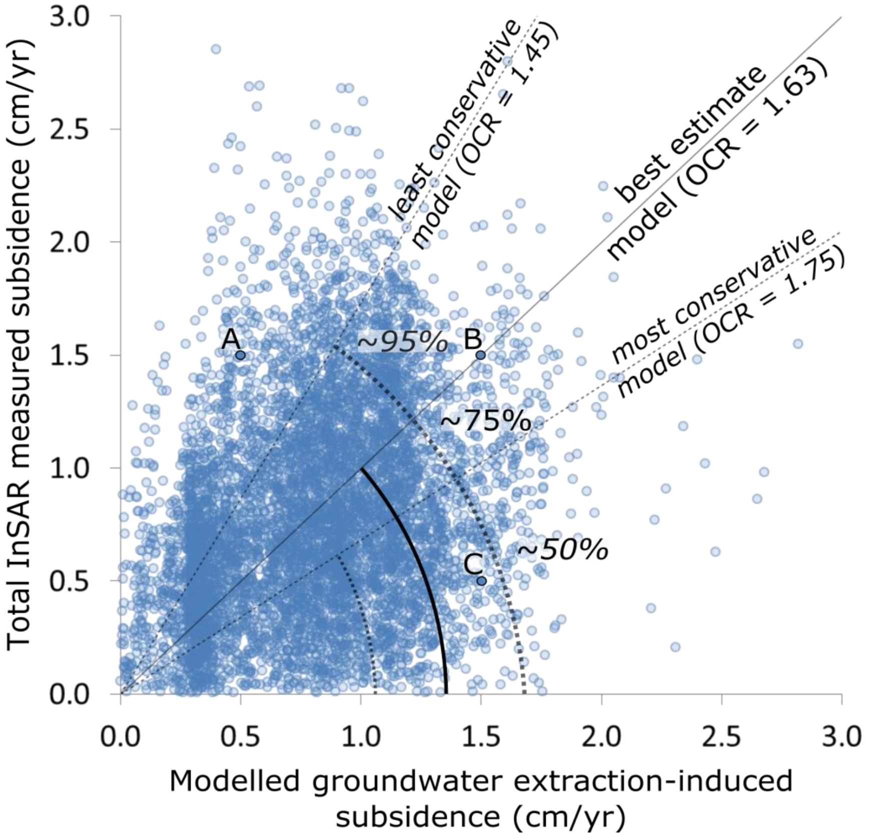

Figure S7 Fit between modelled groundwater extraction-induced subsidence and InSAR-measured subsidence for the MKD (Erban et al 2014) upscaled to model resolution for the best estimate model. Linear fits (y=1x; modelled equals measured subsidence) is shown for the best estimate model (overconsolidation ratio (OCR) of 1.63) and, respectively, for the least and most conservative models with OCRs of 1.45 and 1.75 (data is not shown in this figure). The best estimate model has a rather low cross-correlation (r) of 0.28 which is to be expected as total InSAR measured subsidence and groundwater extraction induced subsidence do not correlate in absolute values (discussed in the main manuscript section: Subsidence in the Mekong Delta). A relative correlation between the two is present, shown by a Spearman's rank-order correlation (ρ) of 0.73. Subsidence rates are in average annual rates over the period 2006-2010.

S8. Data resampling and calculating statistics

S6.1 Resampling InSAR-measured subsidence rates

The spatial resolution of the InSAR-measured subsidence rates as presented by Erban *et al* (2014) (300 m^2) differs from the modelled subsidence cell size (1 km^2). To enable direct comparison, the InSAR-measured subsidence rates were resampled using a median bilinear interpolation creating a weighted value to match the model cell size and raster position.

S6.2 Calculation of spatial statistics on model results

When MKD is explicitly mentioned in the text, this means that model and InSAR measurements outside of the MKD, i.e. HCMC province and Cambodia, are not included in the analysis. As the extraction database does not provide a full spatial coverage of the delta, values of reported average subsidence caused by groundwater extraction are calculated only for parts of the MKD inside a 5 km radius of an extraction with extraction amounts exceeding 5 $m^3/km^2/day$ (Fig S4b). Average subsidence values reported for HCMC were calculated over the area for which >50 cm of total subsidence in the best estimate model was calculated (Fig. 6, main text).

References

Bakr M, de Lange G and Toan D N 2013 *Ho Chi Minh City Flood and Inundation Management Final Report Volume 3 : Institutional Development and Capacity Building Needs and New Partnership* vol 3

Carlson D 2000 Estimate of Vertical Anisotropy of Hydraulic Conductivity for Northern Louisiana Aquifers from Grain-Size Data 32–42

DWRPIS 2010 *Report on the results of the National Groundwater Monitoring Network for Nam Bo Plain. Division of Water Resources Planning and Investigation for the South of Vietnam (DWRPIS)*

Erban L E and Gorelick S M 2016 Closing the irrigation deficit in Cambodia: Implications for transboundary impacts on groundwater and Mekong River flow *J. Hydrol.* **535** 85–92 Online: http://linkinghub.elsevier.com/retrieve/pii/S0022169416300129

Erban L E, Gorelick S M and Zebker H A 2014 Groundwater extraction, land subsidence, and sea-level rise in the Mekong Delta, Vietnam *Environ. Res. Lett.* **9** 84010 Online: http://iopscience.iop.org/1748-9326/9/8/084010%5Cnhttp://stacks.iop.org/1748-9326/9/i=8/a=084010?key=crossref.b639ea338e342899358515e74a86f960

Gunduz Z and Arman H 2007 Possible Relationships Between Compression and Recompression Indices of a Low – Plasticity *Arab. J. Sci. Eng.* **32** 179–90

Higashi T, Ohtsubo M, Hiyama H, Kanayama M and Akaboshi K 2002 A consideration on the compression index of Ariake clay (in Japanese) *The 83th Congress of Kyushu Branch, Japanese Society of Irrigation, Drainage and Reclamation Engineering* pp 215–6

Higgins S, Overeem I, Tanaka A and Syvitski J P M 2013 Land subsidence at aquaculture facilities in the Yellow River delta, China *Geophys. Res. Lett.* **40** 3898–902 Online: http://doi.wiley.com/10.1002/grl.50758

Ladd C C 1977 Stress - deformation and strength characteristics: state of the art report *Proc. 9th ICSMFE* **4** 421–94

Luong V V 2008 *Studying the rainfall, temperature and moisture change in Mekong Delta. Scientific research - Ministry of Science and Technology*

Mesri G and Godlewski P M 1977 Time-and stress-compressibility interrelationship *J. Geotech. Geoenvironmental Eng.* **103**

Minh D, Van Trung L and Toan T 2015 Mapping Ground Subsidence Phenomena in Ho Chi Minh City through the Radar Interferometry Technique Using ALOS PALSAR Data *Remote Sens.* **7** 8543–62 Online: http://www.mdpi.com/2072-4292/7/7/8543/

NNI 2012 *NEN9997-1+C1: Geotechnical design of structures; Part 1: General rules (in Dutch). Nederlands Normalisatie Instituut (Dutch Normalization Institute)*

Ohtsubo M, Higashi T and Kanayama M 2007 Depositional geochemistry and geotechnical properties of marine clays in the Ariake Bay area , Japan *Characterisation and Engineering Properties of Natural Soils, Two Volume Set: Proceedings of the Second International Workshop on Characterisation and Engineering Properties of Natural Soils, Singapore, 29 November-1 December* pp 1893–937

Post V E A, Groen J, Kooi H, Person M, Ge S and Edmunds W M 2013 Offshore fresh groundwater reserves as a global phenomenon *Nature* **504** 71–8 Online: http://dx.doi.org/10.1038/nature12858

Thoang T T and Giao P H 2015 Subsurface characterization and prediction of land subsidence for HCM City, Vietnam *Eng. Geol.* **199** 107–24 Online: http://dx.doi.org/10.1016/j.enggeo.2015.10.009

Toan D M and Nu N T 2013 Studying on the engineering geological characteristics of Middle-Upper Holocene formation (in Vietnamese, English Summary) *Tap chi GIA CHAT, loat A* **so 333** 47–56

Vermeulen P, Hong N, Dinh N and Nam G 2013 Groundwater modeling for the Mekong Delta using iMOD *20th Int. Congr. Model. Simultation* 2499–505 Online: http://www.mssanz.org.au/modsim2013/L4/vermeulen.pdf

Widodo S and Ibrahim A 2012 Estimation of primary compression index (CC) using physical properties of Pontianak soft clay *Int. J. Eng. Res.* **2** 2232–6
